# Vitamin D deficiency increases the risk of bacterial vaginosis during pregnancy: Evidence from a meta-analysis based on observational studies

**DOI:** 10.3389/fnut.2022.1016592

**Published:** 2022-11-22

**Authors:** Lirong Ma, Zhuoran Zhang, Liyang Li, Lijie Zhang, Zhijuan Lin, Hao Qin

**Affiliations:** ^1^School of Public Health, Weifang Medical University, Weifang, Shandong, China; ^2^School of Basic Medicine, Weifang Medical University, Weifang, Shandong, China; ^3^Key Lab for Immunology in Universities of Shandong Province, Weifang Medical University, Weifang, Shandong, China

**Keywords:** bacterial vaginosis, vitamin D deficiency, pregnant women, infections in pregnancy, observational study, meta-analysis

## Abstract

**Background:**

Bacterial vaginosis (BV) is the most common microbiological syndrome in women of childbearing age, causing numerous adverse health issues in pregnant women. Several observational studies have discussed the association between vitamin D deficiency and the risk of BV during pregnancy, but the results were inconclusive. Therefore, this meta-analysis aimed to explore the association between vitamin D deficiency and BV risk in pregnant women.

**Materials and methods:**

We searched four databases, including PubMed, Embase, Cochrane Library, and Web of Science, from their inception to July 2022. Pooled odds ratios (OR) with corresponding 95% confidence intervals (CI) were estimated using random effects models. Additionally, we conducted subgroup analyses to identify the potential sources of between-study heterogeneity. Sensitivity analysis was performed using the method of exclusion, one study at a time. Publication bias was examined using Egger’s test and funnel plot.

**Results:**

A total of 14 studies from 13 articles including 4,793 participants were eligible for this meta-analysis. The outcome showed that vitamin D deficiency may increase the risk of BV during pregnancy by 54% (OR, 1.54; 95% CI, 1.25–1.91; *P* < 0.001). In subgroup analyses, positive associations were also found in studies that were: conducted in black women (OR, 1.56; 95% CI, 0.98–2.48; *P* = 0.060), focused on the first trimester of pregnancy (OR, 2.22; 95% CI, 1.35–3.64; *P* = 0.002), of high quality (OR, 3.05; 95% CI, 1.26–7.41; *P* = 0.014), and adjusted for confounders (OR, 1.28; 95% CI, 1.06–1.55; *P* = 0.012). Sensitivity analysis reported that BV risk during pregnancy resulting from vitamin D deficiency increased by 157% (OR, 2.57; 95% CI, 1.50–4.42; *P* = 0.001) when removing the first two high-weight studies. Publication bias was observed using Egger’s test (*t* = 3.43, *P* = 0.005) and a visual funnel plot.

**Conclusion:**

This meta-analysis showed that vitamin D deficiency is positively associated with the risk of BV during pregnancy. Further high-quality prospective cohort studies are needed to determine whether vitamin D intake reduces the prevalence of BV in pregnant women.

## Introduction

Bacterial vaginosis (BV), the most common vaginal infection among women of childbearing age, is characterized by the disruption of vaginal flora consisting of dominant physiologic *Lactobacillus* species to pathologic anaerobic and facultative bacterial species, such as *Gardnerella vaginalis, Prevotella bivia*, and *Atopobium vaginae* (1). The estimated prevalence of BV in the general population is between 23 and 26% worldwide and reaches up to 33 and 31% in black and Hispanic women, respectively (2). Pregnant women may be more susceptible to BV than the general population, particularly during early pregnancy (3). Aside from causing urogenital infections and pelvic inflammatory diseases, having BV during pregnancy may lead to numerous adverse obstetric outcomes, such as preterm birth, late miscarriage, intrauterine fetal death, chorioamnionitis, and low birth weight (4, 5). Additionally, the treatment of symptomatic BV leads to an economic burden that amounts to approximately $4.8 billion worldwide (2). However, the pathogenesis of BV remains poorly understood (5). Given its multiple adverse health outcomes, high recurrence rate, and enormous medical costs, it is pertinent to identify the associated risk factors for this condition, particularly among pregnant women, since this is the first step to preventing infection. Many factors play a role in the development of this infection, such as a higher number of sexual partners, young age at first intercourse, regular vaginal douching, and cigarette smoking (5, 6).

In addition, low vitamin D levels during pregnancy may increase the occurrence of BV (7). Vitamin D not only plays a crucial role in bone development, but also in immune-modulation, which includes triggering anti-inflammatory responses, such as cathelicidin expression and reducing pro-inflammatory cytokine production (e.g., IL-1β) (8). It was estimated that the prevalence of Vitamin D deficiency was about 30% in children and adults worldwide (9, 10). Considering that vitamin D deficiency is highly prevalent among pregnant women worldwide (10), the role of vitamin D in BV risk needs to be examined. To date, there are only a handful of epidemiological studies that have explored the relationship between vitamin D deficiency and the risk of BV in pregnancy (11–23).

However, despite the growing body of research on the relationship between vitamin D and BV risk in pregnancy, the existing literature has yielded inconsistent results. Some studies have reported a positive association between vitamin D deficiency and the occurrence of BV during pregnancy (11–18). Conversely, other studies failed to confirm this association (19–23). Although the association between vitamin D levels and the risk of BV during pregnancy has been mentioned in some systematic review articles (24, 25), these studies have only included a few studies that fulfilled the minimum requirement for meta-analysis. To address this controversial issue further, we gathered relevant data for a meta-analysis that quantitatively assesses the relationship between vitamin D deficiency and BV risk during pregnancy.

## Materials and methods

### Search strategy

We carried out an overall literature search from inception up to July 2022 using four databases: PubMed, Embase, Cochrane Library, and Web of Science. A search strategy was developed involving a combination of keywords and MeSH (Medical Subject Headings) or Emtree terms with boolean operators “OR” and “AND” in all databases to enhance the probability of obtaining related studies. The complete electronic search strategy is presented in Supplementary Table 1.

### Inclusion criteria

For studies included in this meta-analysis, the following criteria were met: (1) original papers published in English; (2) the exposure of interest was vitamin D measurement during pregnancy; (3) the outcome of interest was BV; (4) odds ratio (OR), relative risk (RR), or hazard ratio with 95% confidence interval (CI) (or data/figure to estimate them); (5) observational studies (cohort, case-control, or cross-sectional design); and (6) the most recent and complete study was selected if data from the same population had been published more than once. Meanwhile, if effect sizes were available for meta-analysis in the conference paper, these should also be extracted. In addition, the reference lists of the retrieved articles were carefully examined to avoid missing any relevant literature. All retrieved studies were carefully and independently reviewed by three investigators to determine whether an individual study met the inclusion criteria. If the three investigators (LM, ZZ, and LL) were disputable regarding the eligibility of an article, they were resolved by having a consensus or consultation with a fourth investigator (HQ).

### Data extraction

During the process of literature screening, the title and abstract were reviewed first, before the full texts were further read to determine whether they should be included in the analysis. The extracted data that were obtained included the following details: the first author’s last name, year of publication, country where the study was conducted, research type, sample size, mean age or age range of participants, gestational age when vitamin D was measured, vitamin D and BV determination methods, threshold of vitamin D deficiency, and adjusted confounding factors, the ORs (we used OR to represent the effect size for simplicity) with corresponding CIs of BV for vitamin D deficiency. When multiple ORs (95% CIs) were reported, we only extracted the effect estimates after adjusting for most confounders. In addition, as pregnant women in early stage may be more susceptible to BV and tend to suffer more from BV-induced adverse pregnancy outcomes than women in middle and late pregnancy (3–5), we preferentially used the OR (95% CI) of BV risk for vitamin D deficiency during early pregnancy in individual studies to calculate the pooled effect estimate if several ORs (95% CIs) were provided at different gestational ages (e.g., early, middle, and late pregnancy).

### Quality assessment

The Newcastle-Ottawa Scale (NOS), a scoring system developed to assess the risk of bias, was used to evaluate the quality of the studies. The NOS covers three domains: selection, comparability, and exposure/outcome. A study can be awarded a maximum of one star for each numbered item within the selection and exposure categories. A maximum of two stars can be assigned for comparability. Each star represents one point; thus, the maximum possible score is nine. Generally, a total score of seven or more indicates high quality and a score of less than seven represents low quality.

### Statistical analyses

To determine the strength of the association between vitamin D deficiency and the risk of BV during pregnancy, the DerSimonian and Laird random effects model was used to calculate the pooled OR (95% CI) in view of inevitable between-study variance (26). Between-study heterogeneity was assessed using the I^2^ statistic (I^2^ values of 0—25%, 25–50%, 50–75%, and 75–100% indicate no, low, medium, and high heterogeneity, respectively) (27). To explore the possible sources of heterogeneity, subgroup analyses were performed to examine the role of potential confounding factors, such as study type, geographic location where studies were conducted, race, gestational age, vitamin D assay methods, adjustment for confounders, study quality and climate characteristic of area of included paper. Sensitivity analyses were conducted, with one study excluded at a time, to assess the stability of the pooled OR (95% CI). Additionally, the Egger regression asymmetry test and visual inspection of funnel plots were used to evaluate publication bias (28).

We used Stata 14.2 software (Stata Corporation, College Station, TX, USA) to perform data analyses. All 2-tailed *P-*values < 0.05 were considered statistically significant.

## Results

**FIGURE 1:**
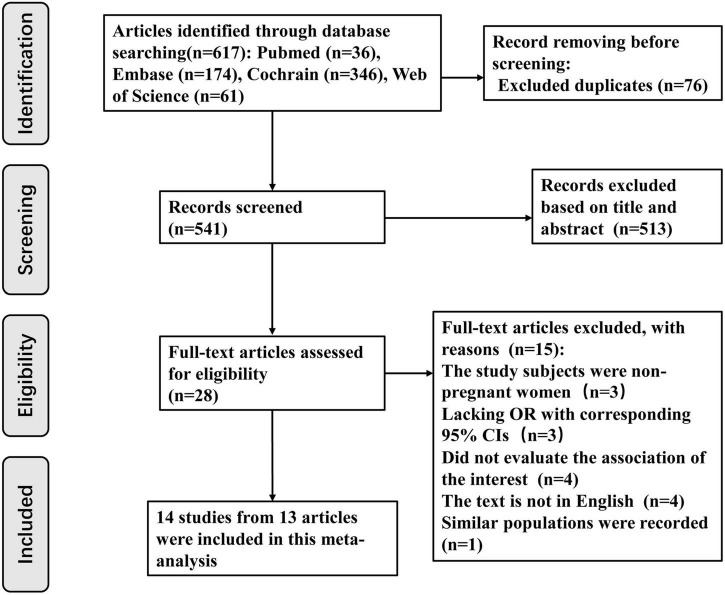
Flow chart of the selection of studies included in this meta-analysis.

Initially, the database search allowed the investigators collect 617 articles (PubMed 36, Embase 174, Cochrane Library 346, and Web of Science 61). A total of 541 articles were examined through their titles and abstracts after excluding 76 duplicates. Subsequently, 513 articles were removed because they explicitly did not meet the inclusion criteria. From the remaining 28 articles, which were carefully reviewed to assess if they fit the criteria, 15 were rejected for the following reasons: 3 articles focused on non-pregnant women (29–31); 3 articles lacked ORs and corresponding 95% CIs, which could not be obtained from the available data (7, 32, 33). Among these three studies (7, 32, 33), two studies supported that vitamin D deficiency in the first (33) and second (7) trimesters of pregnancy increased BV occurrence, respectively, while one study (32) considered that neither vitamin D deficiency in early pregnancy nor supplementation reduced BV risk during pregnancy. Four studies did not quantitatively evaluate the association between vitamin D deficiency and BV risk (34–37). Among these four studies (34–37), one study was a letter to the editor (34), one was a review (35), one focused on the association between vitamin D status and the vaginal microbiome (36), and one study concentrated on complications of gestation (37). Four articles were not published in English (38–41). One study used a similar population, with the most recent studies included (42). Fourteen studies from 13 articles published from 2009 to 2021 were eligible for this meta-analysis. Detailed information regarding the literature retrieval process is shown in Figure 1.

### Baseline characteristics

Among the included studies, eight studies were carried out in North America 8 (11–13, 16, 17, 21, 23), 2 in Asia (15, 20), 3 in Europe (14, 18, 22) and 1 in Africa (19). Regarding the study type, one study had a case-control design (10), four had a cohort design (14, 18, 19, 21), and nine had a cross-sectional design (11, 13, 15–17, 20, 22, 23). Regarding the adjustment for confounding factors, 10 were adjusted (11–13, 15, 16, 19–21, 23) and 4 unadjusted (14, 17, 18, 22). With respect to BV determination methods, 10 studies were based on the Nugent score of Gram staining (11–13, 15–17, 19, 21, 22), one used 16S rRNA sequencing technology (23), and three did not report measurement methods (14, 18, 20). For the timing of blood sample collection to measure vitamin D levels, 10 studies focused on the first trimester (11, 13, 15–19, 21, 22), 2 focused on the second trimester (20, 22), and 3 focused on the third trimester (12, 14, 21). According to the scores based on the Newcastle-Ottawa scale, five were considered to be of high quality (11, 13, 15, 17, 19), and nine were classified as low quality (12, 14, 16, 18, 20–23). As for climate characteristic of area of included paper, 12 studies were belonged to temperate zone (11–18, 21–23), two were categorized as tropical zone (19, 20). The baseline characteristics of the included studies are presented in Table 1.

**TABLE 1:** Baseline characteristics of the included studies.

Ref.	Country	Population	Study design	Age (years)	Gestational age (weeks)	No. of Participants (case)	Measurement method of vitamin D	Determination method of BV	Threshold of vitamin D deficiency	Evaluation of vitamin D	OR (95% CI) for vitamin D deficiency	Adjustment for covariates	Quality assessment
Bodnar et al. (11)	United States	Black and White	Cross-sectional	20–29	<16	469 (192)	RIA	Nugent score of Gram staining	<20 nmol/L	25-hydroxy-vitamin D	Total population: 1.65 (1.01, 2.69); black women: 1.47 (1.02, 2.13); white women: 1.09 (0.62, 1.92)	Sexually transmitted diseases	7
Dunlop et al. (12)	United States	Non-Hispanic black, Non-Hispanic white	Case-control	24.14 ± 6.04	31–37	160 (14)	ELISA	Nugent score of Gram staining	<12 ng/ml	25-hydroxy-vitamin D	Total population: 5.11 (1.19, 21.97)	Race, age, smoking status, BMI, gestational age at delivery, payor source	6
Hensel et al. (13)	United States	Non-Hispanic white, Non-Hispanic black, Mexican American	Cross-sectional	14–49	<13	440 (NR)	Microbiological method	Nugent score of Gram staining	<30 ng/ml	25-hydroxy-vitamin D	Total population: 2.87 (1.13, 7.28)	Age, race, education, poverty index, marital status, age at first sex, number lifetime partners, ever have female sex partner, unprotected sex, pregnancy status, oral contraception use, douching frequency last six months, cotinine level, BMI	7
Skowrońska et al. (14)	Poland	Polish	Cohort	30.5 ± 4.9	28–40	102 (NR)	ECLIA	NR	<20 ng/ml	Vitamin D supplement	White women: 10.77 (2.09, 55.40)	NR	4
Rahmanpour et al. (15)	Iran	Persian	Cross-sectional	NR	<20	204 (55)	Microbiological method	Nugent score of Gram staining	<20 nmol/L	25-hydroxy-vitamin D	White women: 16.30 (6.00, 45.50)	BMI, maternal age	7
Turner et al. (19)	Zimbabwe	Zimbabwean	Cohort	22–28	<13	141 (38)	RIA	Nugent score of Gram staining	<30 ng/ml	25-hydroxy-vitamin D	Black women: 0.88 (0.51, 1.54)	Age, education, parity, HSV-2 status, circumcision status of primary male partner, sex in the last three months, vaginal hygiene habits, sexual frequency, condom use, number of male sex partners	7
Tabatabaei et al. (16)	Canada	Montrealer	Cross-sectional	NR	8–14	433 (NR)	LC-MS	Nugent score of Gram staining	<50 nmol/L	25-hydroxy-vitamin D	Ethnic minority (black women): 5.60 (1.58, 19.84); non-ethnic minority (white women): 1.31 (0.73, 2.35)	Season of conception, age, pre-pregnancy BMI, parity, marital status, smoking, education, present history of sexually transmitted disease	5
Powell et al. (17)	United States	African American	Cross-sectional	NR	8–12	245 (63)	Microbiological method	Nugent score of Gram staining	<40 ng/ml	Vitamin D supplement	Black women: 5.26 (3.20, 12.82)	NR	7
Lee et al. (20)	Malaysia	Malay, Chinese, Indian, other ethnicity	Cross-sectional	30.0 ± 4.36	>37	575 (13)	HPLC	NR	<20 ng/ml	25-hydroxy-vitamin D	Total population: 1.01 (0.95, 1.08)	Maternal age, BMI	4
Dunlop et al. (21)	United States	African American	Cohort	24.3 ± 4.3	8–14	137 (57)	CLIA	Nugent score of Gram staining	<20 ng/ml	Total and free 25 (OH)D	Black women: 1.04 (0.99, 1.10) First trimester: 1.04 (0.99, 1.10); last trimester: 1.06 (1.01, 1.12)	Maternal age, parity, insurance status, first prenatal BMI, gestational age of visit, receipt of antibiotics in the month prior to the visit	5
Christoph et al. (22)	Switzerland	European, Northern Africa, Middle East, South West Asia, Sub-Saharan Africa, Indian	Cross-sectional	22–38	8–16	1153 (36)	CLIA	Amsel criteria and Nugent scoring	<25 nmol/L	Vitamin D supplements	Total population: 0.69 (0.27, 1.52)	NR	4
Maliar (18)	Ukraine	Ukraninian	Cohort	25.1 ± 2.6	10–12	100 (19)	ECLIA	Nugent score of Gram staining	<30ng/ml	25-hydroxy-vitamin D	White women: 4.93 (1.50, 16.16)	NR	5
Rosen et al. (23)	United States	Black and White	Cross-sectional	26.6 ± 6.9	24–29	634 (76)	FFQ	16S rRNA sequencing technology	NR	Dietary vitamin D	Total population: 0.83 (0.51, 1.37); black women: 0.83 (0.38, 1.85); white women: 0.88 (0.47, 1.67)	Race, age, parity, BMI, maternal stress	5

Ref., reference; CLIA, chemiluminescent immunoassay; ECLIA, electrochemical luminescence immunoassay; ELISA, enzyme-linked immunosorbent assay; FFQ, food frequency questionnaire; HPLC, high-performance liquid chromatography; LC-MS, liquid chromatography-mass spectrometry; RIA, radioimmunoassay; NR, not reported; OR, odds ratio; CI, confidence interval; BV, bacterial vaginosis; BMI, body mass index; HSV-2, herpes simplex virus type 2.

### Quantitative synthesis

**FIGURE 2:**
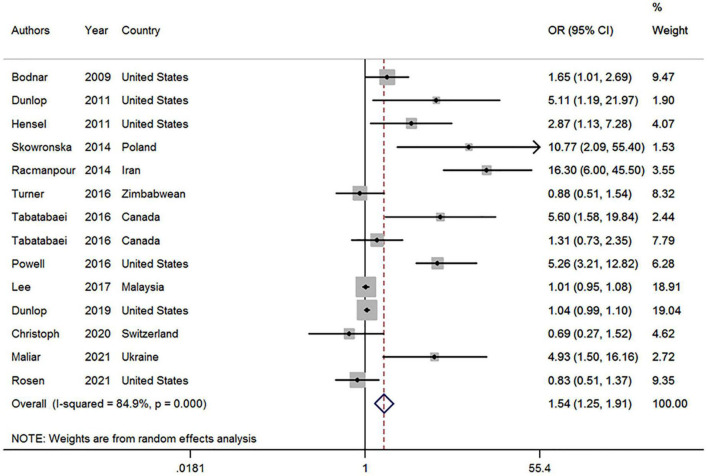
Forest plot of included studies on the association between vitamin D deficiency and bacterial vaginosis risk. OR, odds ratio; CI, confidence interval. The size of the grey box is positively proportional to the weight assigned to each study, which is inversely proportional to the SE of the OR. The horizontal lines represent the 95% CI.

This meta-analysis used data from 14 studies in 13 articles covering 4,793 participants to assess the association between vitamin D deficiency and the risk of BV during pregnancy. Of the 14 studies, six reported no relationship between vitamin D deficiency and BV prevalence during pregnancy, while eight showed a positive association between the two. Our results showed a positive association between vitamin D deficiency and the risk of BV during pregnancy (OR, 1.54; 95% CI, 1.25–1.91, *P* < 0.001; I^2^ = 84.9%, P_heterogeneity_ < 0.01; Figure 2).

### Subgroup analyses

In view of the high heterogeneity among the included studies, subgroup analyses were performed to examine possible confounders. As shown in Table 2, the pooled OR (95% CI) for subgroups stratified by study type was 1.78 (0.87–3.64), 5.11 (1.19–21.96), and 2.04 (1.23–3.39) in cohort, case-control, and cross-sectional studies, respectively. The combined OR (95% CI) for subgroups by gestation age was 2.22 (1.35–3.64) in the first trimester, 1.01 (0.95–1.08) in the second trimester, and 3.31 (0.69–15.94) in the third trimester. The pooled OR (95% CI) was 1.56 (0.98–2.48) for studies performed in the black population, 1.92 (1.22–3.03) for studies executed in North America, 2.51 (1.55, 4.06) for studies conducted in temperate zone, and 2.20 (1.34–3.61) for studies using Nugent Score of Gram staining. Additionally, the pooled ORs (95% CIs) in high-quality studies and studies adjusted for confounders were 3.05 (1.26–7.41) and 1.28 (1.06–1.55), respectively.

**TABLE 2:** Pooled ORs of subgroup analyses for the association between Vitamin D deficiency and the risk of bacterial vaginosis (BV).

Subgroups	No. of studies (ref.)	Pooled ORs (95% CIs)	*P*-values for pooled ORs	*P*-values for subgroup differences	Study heterogeneity	
					I^2^ (%)	*P*-value
All studies	14 (11–23)	1.54 (1.25–1.91)	<0.001	–	84.9	<0.001
**Study design**						
Cohort	4 (14, 18, 19, 21)	1.78 (0.87–3.64)	0.117	0.957	79.6	0.002
Case-control	1 (12)	5.11 (1.19–21.96)	0.028		–	–
Cross-sectional	9 (11, 13, 15–17, 20, 21, 23)	2.04 (1.23–3.39)	0.006		88.1	<0.001
**Geographic location**						
North America	8 (11–13, 16, 17, 21, 23)	1.92 (1.22–3.03)	0.005	0.794	82.9	<0.001
Asia	2 (15, 20)	3.87 (0.25–58.86)	0.329		96.5	<0.001
Europe	3 (14, 18, 22)	3.01 (0.55–16.49)	0.203		83.5	0.002
Africa	1 (19)	0.88 (0.51–1.53)	0.650		–	–
**Race/Ethnicity**						
Total population	6 (11–13, 20, 22, 23)	1.25 (0.86–1.82)	0.234	0.265	65.7	0.012
Black women	6 (11, 16, 17, 19, 21, 23)	1.56 (0.98–2.48)	0.060		84.1	<0.001
White women	6 (11, 14–16, 18, 23)	2.77 (1.16–6.59)	0.021		85.5	<0.001
**Trimester of blood collection**						
First	10 (11, 13, 15–19, 21, 22)	2.22 (1.35–3.64)	0.002	0.974	87.4	<0.001
Second	2 (20, 23)	1.01 (0.95–1.08)	0.778		0.0	0.435
Last	3 (12, 14, 21)	3.31 (0.69–15.94)	0.135		83.5	0.002
**Vitamin D assay methods**						
Instrumental method	10 (11, 12, 14, 16, 18–22)	1.17 (0.99–1.38)	0.060	0.759	71.6	<0.001
Microbiological method	3 (13, 15, 17)	6.09 (2.50–14.85)	<0.001		68.1	0.044
Food frequency questionnaire	1 (23)	0.83 (0.51–1.36)	0.462		–	–
**Determination of BV**						
Nugent score of Gram staining	10 (11–13, 15–17, 19, 21, 22)	2.20 (1.34–3.61)	0.002	0.557	87.0	<0.001
16srDNA sequencing technology	1 (23)	0.83 (0.51–1.36)	0.462		–	–
NR	3 (14, 18, 20)	3.24 (0.70–15.13)	0.134		86.4	0.001
**Adjusted for confound factors**						
Adjusted	10 (11–13, 15, 16, 19–21, 23)	1.28 (1.06–1.55)	0.012	0.386	82.0	<0.001
Unadjusted	4 (14, 17, 18, 22)	3.44 (1.03–11.44)	0.044		82.1	0.001
**Study quality**						
High quality	5 (11, 13, 15, 17, 19)	3.05 (1.26–7.41)	0.014	0.382	88.0	<0.001
Low quality	9 (12, 14, 16, 18, 20–23)	1.12 (0.95–1.32)	0.196		72.1	<0.001
**Climate characteristic**						
Temperate zone	12 (11–18, 21–23)	2.51 (1.55, 4.06)	<0.001	0.166	86.9	<0.001
Tropical zone	2 (19, 20)	1.01 (0.95, 1.08)	0.744		0.0	0.620

Ref., reference; OR, odds ratio; CI, confidence interval; BV, bacterial vaginosis; NR, not reported.

### Sensitivity analysis

To assess the robustness of our meta-analysis, a sensitivity analysis was conducted by excluding one study at a time (Supplementary Figure 1). The findings of the sensitivity analysis suggest that the first two high-weight studies considerably lowered the pooled effect size (18, 19). After removing the two studies, the pooled OR (95% CI) of BV prevalence for vitamin D deficiency was from 1.54 (1.25–1.91, *P* < 0.001) to 2.57 (1.50–4.42, *P* = 0.001) (Supplementary Figure 2) and was relatively stable (Supplementary Figure 3).

### Publication bias

Publication bias was observed using Egger’s test (*t* = 3.43, *P* = 0.005) and visual inspection of the funnel plot for the effect of vitamin D deficiency on BV risk (Supplementary Figure 4).

## Discussion

The current meta-analysis quantitatively evaluated the association between vitamin D deficiency and BV risk in pregnant women. In contrast, previous published reviews mainly narratively described the findings of original studies, partly due to limiting sample size (each review covering three studies) (24, 25). This meta-analysis, including 14 studies from 13 articles covering 4,793 participants, showed that vitamin D deficiency could increase the risk of BV by 54% during pregnancy. More specifically, for vitamin D deficiency in the first trimester and for black women, the BV risks were elevated up to 122 and 56%, respectively. Furthermore, a similar trend was found in the high-quality (OR, 3.05; 95% CI, 1.26–7.41; *P* = 0.014), adjustment for confounders (OR, 1.28; 95% CI, 1.06–1.55; *P* = 0.012), and cohort study (OR, 1.78; 95% CI, 0.87–3.64; *P* = 0.117) subgroups. In addition, our findings are partly supported by a randomized clinical trial by Taheri et al., who reported that the treatment of vitamin D deficiency might eliminate asymptomatic BV in non-pregnant women (43). Thus, according to the recommendations from Institute of Medicine, pregnant women had better ingest on average 600 IUs of vitamin D daily and maintain the serum vitamin D at least 30 ng/mL (10).

The exact biological mechanism by which vitamin D deficiency increases susceptibility to BV is not yet well established. To date, several possible biological pathways have been proposed to elucidate the role of vitamin D in the prevalence of BV. First, vitamin D is implicated in the regulation of the proliferation and differentiation of various cells (44), particularly in stratified squamous epithelium, such as the vaginal epithelium (45). One of the mechanisms underlying this may be that vitamin D triggers the VDR (vitamin D receptor)/p-RhoA (ras homolog gene family)/p-Ezrin (cell junction proteins) pathway, which may increase cell-to-cell junctions of the vaginal epithelium and decrease the PH value of the vaginal microbial environment (46, 47). Additionally, vitamin D deficiency may induce vaginal atrophy, decrease barrier function, and increase BV risk.

Second, vitamin D is linked to diverse immunomodulatory actions, including the enhancement of the innate immune system and regulation of the adaptive immune responses, through binding to VDRs expressed by a number of different immune cell subsets (44). On the one hand, with the activation of toll-like receptors, vitamin D and VDR binding enhances the antimicrobial activities of key innate immunocytes, such as neutrophils, monocytes, and macrophages. These effects are principally mediated by up-regulating the synthesis of antimicrobial peptides, such as cathelicidins and beta-defensins, which could prevent and control invasive bacterial infections and increase genital tract immune capacity (44, 48–50). In contrast, VDR ligation by vitamin D enhances anti-inflammatory cytokine production (51, 52), such as interleukin-4 (IL-4) and interleukin-10 (IL-10), and inhibits the expression of pro-inflammatory cytokines (44), such as interleukin-1β (IL-1β), interleukin-6 (IL-6), interleukin-12 (IL-12), tumor necrosis factor-alpha (TNF-α), and the development of pro-inflammatory T helper 1 (Th1) and T helper 17 (Th17) cells (53). Vitamin D also inhibits the production of interleukin-2 (IL-2), which is essential for lymphocyte clonal expansion and interferon-gamma (IFN-γ) (54). Thus, given the decrease of antimicrobial peptide synthesis and anti-inflammatory cytokine production, and the increase in pro-inflammatory cytokine expression, vitamin D deficiency may promote the occurrence of BV.

Additionally, vitamin D may play a role in influencing the vaginal microbial environment. By elevating calcium concentration, vitamin D may stimulate insulin secretion and increase glycogen synthesis, which induces glycogen deposition in the vagina (36, 55). A higher concentration of free glycogen in the lower genital tract promotes *Lactobacillus* species colonization, decreases vaginal pH, and inhibits the growth of other bacteria (56). A pilot study including black adolescent women also showed that higher vaginal glycogen levels were positively related to the dominance of *Lactobacillus* (57). Therefore, vitamin D deficiency may alter glucose homeostasis in the vagina and enhance BV prevalence.

In the current study, subgroup analyses stratified by race revealed that vitamin D deficiency might increase BV risk in black women. The potential reason may be that most black women usually suffer from a higher burden of vitamin D deficiency than white women (58). Additionally, vitamin D intake from diet and supplementation for black women is relatively low (59). In contrast, darker skin pigmentation may inhibit conversion from 7-dehydrocholesterol (provitamin D_3_) to pre-calciferol (pre-vitamin D_3_ form) following sun exposure (60). In addition, lifestyle factors such as regular vaginal douching and cigarette smoking, which are known risk factors for BV (5, 6), are likely to differ among races. These findings suggest that race is not an independent factor for BV occurrence. Nonetheless, some studies have reported that race/ethnicity exerts an effect on the diversity and predominance of the vaginal microbiome (61, 62). In addition, it is puzzling that there was statistical association between vitamin D deficiency and BV risk in subgroup analysis on white women. Thus, further studies are needed to clarify the association between race and BV.

### Strengths and limitations

Our study has several strengths. To our knowledge, this is the first meta-analysis in recent years to examine the relationship between vitamin D deficiency and BV risk during pregnancy. Second, based on potential confounders, such as race and gestational age, and adjustment for confounders, various subgroup analyses were performed.

Our study has some limitations. First, the cross-sectional or case-control design used in some original studies limits the establishment of causality due to inevitable recall and selection biases. Second, the studies included in this meta-analysis were biased toward North America and Europe, which might reduce the generalizability of our outcomes. Third, the threshold for vitamin D deficiency defined in the included studies was inconsistent, which may have underestimated the pooled ORs (95% CIs). Fourth, I^2^ values of between-study heterogeneity remained high even though numerous subgroup analyses were conducted, suggesting that unaccounted potential confounders may exist. Thus, we structured a random effects model to minimize the influence of between-study heterogeneity. Fifth, some extracted ORs from the original studies used to calculate combined effect estimates were estimated based on a frequency table or figure, which may lead to unavoidable bias. Sixth, the methods used to measure vitamin D levels and diagnose BV were not entirely consistent, which may have influenced the stability of the results. Seventh, publication bias was observed using Egger’s test and funnel plot. Finally, we were unable to investigate a dose-response relationship between vitamin D levels and BV risk owing to the lack of sufficient data.

## Conclusion

Our meta-analysis, involving 14 studies, showed that vitamin D deficiency contributes to the risk of BV during pregnancy. Most subgroup analyses also supported this finding, especially in studies that were focused on the first trimester of pregnancy, considered high quality, and adjusted for confounders. Considering the high prevalence and adverse health outcomes of vitamin D deficiency and BV, these findings have potential clinical implications. Additional studies, especially large prospective cohort studies in various races, are required to further assess the association between vitamin D deficiency and BV risk.

## Data availability statement

The original contributions presented in this study are included in the article/Supplementary material, further inquiries can be directed to the corresponding authors.

## Author contributions

LM and HQ contributed to conception and design of the study. LM, ZZ, and LL extracted data and wrote the first draft of the manuscript. LM, HQ, and LZ performed the statistical analysis. HQ and ZL reviewed and edited the manuscript. All authors contributed to manuscript revision, read, and approved the submitted version.
