# Supplementary material for: Vitamin D deficiency increases the risk of bacterial vaginosis during pregnancy: Evidence from a meta-analysis based on observational studies
Source: Front Nutr. 2022 Nov 22;9:1016592. doi: 10.3389/fnut.2022.1016592 (PMC9722752; doi:10.3389/fnut.2022.1016592)
Supplement: Supplementary file 1 [file Data_Sheet_2.PDF]

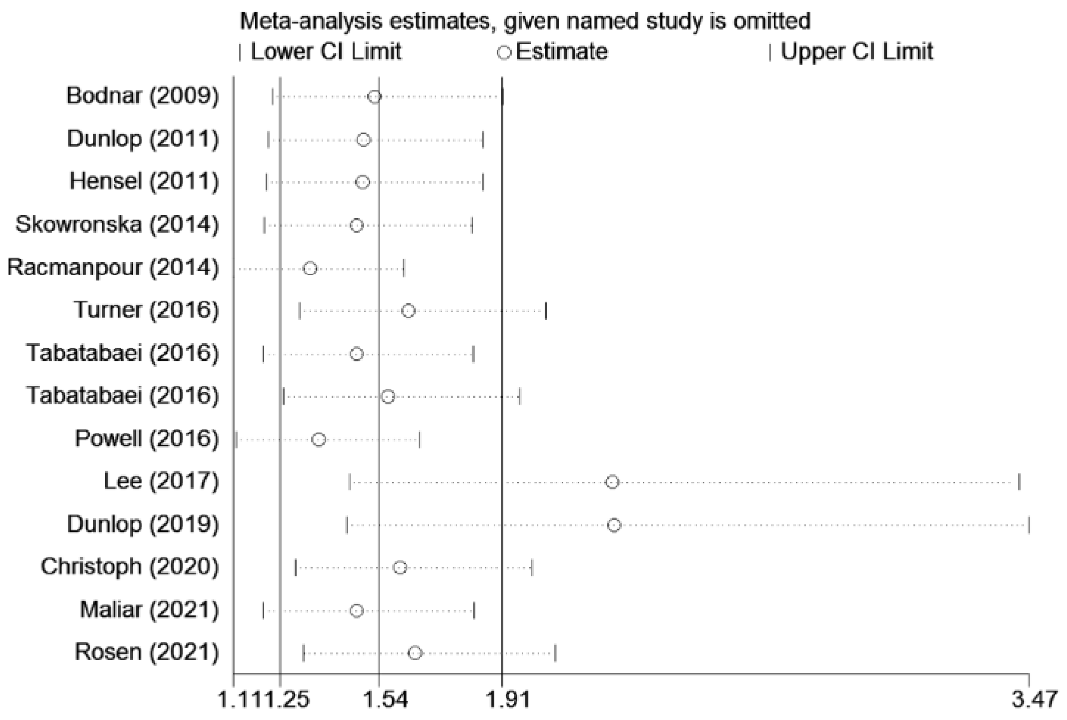

Supplementary Figure 1 Sensitivity analysis using the method of one study excluded at a time. The middle line represents the pooled effect estimates. The bilateral lines represent 95% confidence interval.
