# Supplementary material for: Vitamin D deficiency increases the risk of bacterial vaginosis during pregnancy: Evidence from a meta-analysis based on observational studies
Source: Front Nutr. 2022 Nov 22;9:1016592. doi: 10.3389/fnut.2022.1016592 (PMC9722752; doi:10.3389/fnut.2022.1016592)
Supplement: Supplementary file 2 [file Data_Sheet_3.PDF]

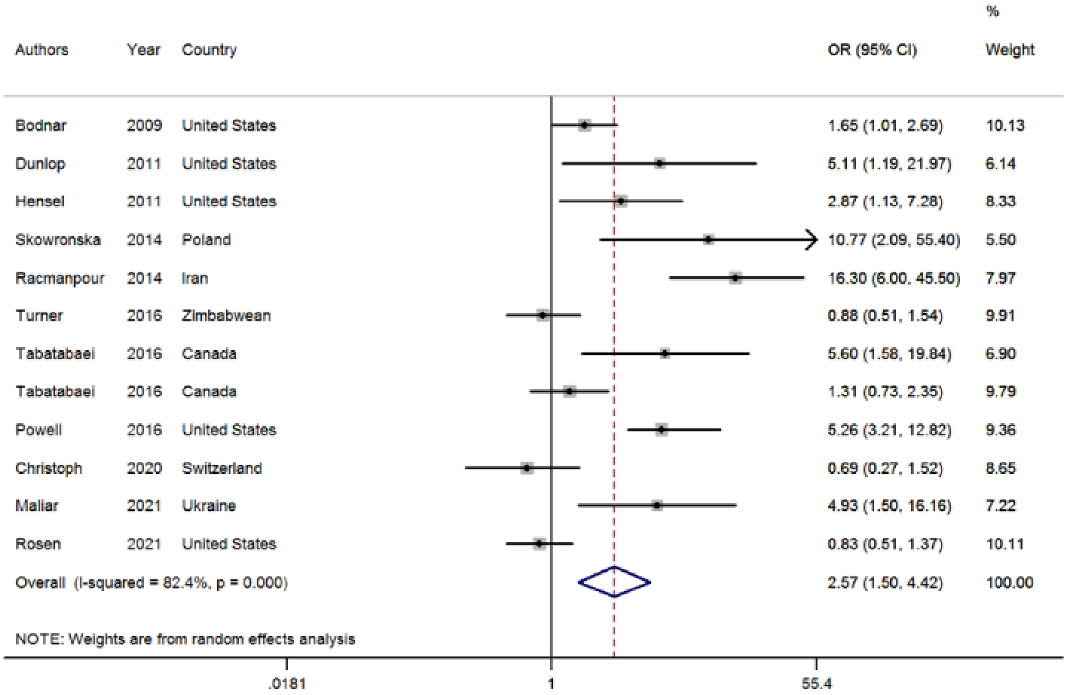

Supplementary Figure 2 Forest plot after removing the first two high-weight studies which considerably influencing the combined effect size. OR, odds ratio; CI, confidence interval. The size of the grey box is positively proportional to the weight assigned to each study, which is inversely proportional to the SE of the OR. The horizontal lines represent the 95% CI.
