# Supplementary figures and images for: Vitamin D deficiency increases the risk of bacterial vaginosis during pregnancy: Evidence from a meta-analysis based on observational studies
Source: Front Nutr. 2022 Nov 22;9:1016592. doi: 10.3389/fnut.2022.1016592 (PMC9722752; doi:10.3389/fnut.2022.1016592)

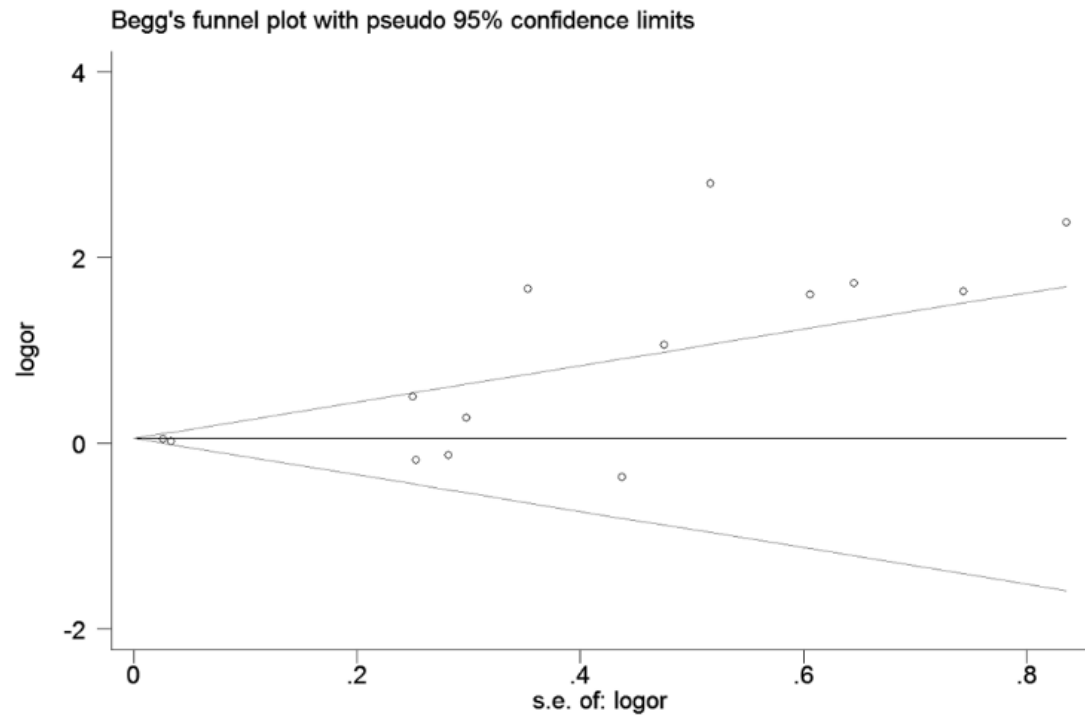

**Supplementary Figure 4** Funnel plot of included 14 studies. Each dot represents a different study.

Supplement: Supplementary file 4 [file Data_Sheet_5.PDF]
