# Supplementary material for: Vitamin D deficiency increases the risk of bacterial vaginosis during pregnancy: Evidence from a meta-analysis based on observational studies
Source: Front Nutr. 2022 Nov 22;9:1016592. doi: 10.3389/fnut.2022.1016592 (PMC9722752; doi:10.3389/fnut.2022.1016592)
Supplement: Supplementary file 5 [file Table_1.DOCX]

Supplementary Table 1 The complete electronic search strategy for PubMed, Embase, Cochrane Library, and Web of Science.

| **Databases** | **Electronic search strategy** |
| --- | --- |
| **Embase** | #1 25hydroxyvitamind:ab,ti OR '25hydroxyergocalciferol':ab,ti OR ergocalciferol:ab,ti OR cholecalciferol:ab,ti OR hydroxycholecalciferol:ab,ti OR calcifediol:ab,ti OR dihydroxycholecalciferol:ab,ti OR '1,25 ( oh ) 2-vitd':ab,ti OR '25 ( oh ) d':ab,ti  #2 'vitamin d'/exp  #3 #1 OR #2  #4 'bacterial vaginitides':ab,ti OR 'vaginitides, bacterial':ab,ti OR vaginitis:ab,ti OR 'vaginitis, nonspecific':ab,ti OR 'nonspecific vaginitis':ab,ti OR 'bacterial vaginoses':ab,ti OR 'vaginoses, bacterial':ab,ti OR 'bacterial vaginitis':ab,ti OR 'vaginitis, bacterial':ab,ti  #5 'vaginitis'/exp  #6 #4 OR #5  #7 #3 AND #6 |
| **Pubmed** | (((((((((("Vaginosis, Bacterial"[Mesh]) OR (Bacterial Vaginitides[Title/Abstract])) OR (Vaginitides, Bacterial[Title/Abstract])) OR (Bacterial Vaginosis[Title/Abstract])) OR (Vaginitis, Nonspecific[Title/Abstract])) OR (Nonspecific Vaginitis[Title/Abstract])) OR (Bacterial Vaginoses[Title/Abstract])) OR (Vaginoses, Bacterial[Title/Abstract])) OR (Bacterial Vaginitis[Title/Abstract])) OR (Vaginitis, Bacterial[Title/Abstract])) AND (((((((((("Vitamin D"[Mesh]) OR (25hydroxyvitaminD[Title/Abstract])) OR (25-hydroxyergocalciferol[Title/Abstract])) OR (25(OH)D[Title/Abstract])) OR (1,25(OH)2-vitD[Title/Abstract])) OR (ergocalciferol[Title/Abstract])) OR (cholecalciferol[Title/Abstract])) OR (hydroxycholecalciferol[Title/Abstract])) OR (calcifediol[Title/Abstract])) OR (dihydroxycholecalciferol[Title/Abstract])) |
| **Web of science** | #1 Vitamin D (Topic) or 25-hydroxyvitaminD (Topic) or 25-hydroxyergocalciferol (Topic) or 25(OH)D (Topic) or 1,25(OH)2-vitD (Topic) or ergocalciferol (Topic) or cholecalciferol (Topic) or hydroxycholecalciferol (Topic) or calcifediol (Topic) or dihydroxycholecalciferol (Topic)  #2 Vaginosis, Bacterial (Topic) or Bacterial Vaginitides (Topic) or Vaginitides, Bacterial (Topic) or Bacterial Vaginosis (Topic) or Vaginitis, Nonspecific (Topic) or Nonspecific Vaginitis (Topic) or Bacterial Vaginoses (Topic) or Vaginoses, Bacterial (Topic) or Bacterial Vaginitis (Topic) or Vaginitis, Bacterial (Topic)  #3 #1 and #2 |
| **Cochrane** | #1 MeSH descriptor: [Vaginosis, Bacterial] explode all trees  #2 Bacterial Vaginosis  #3 Bacterial Vaginoses  #4 Vaginitis, Nonspecific  #5 Vaginitis, Bacterial  #6 Vaginoses, Bacterial  #7 Bacterial Vaginitides  #8 Nonspecfic Vaginitis  #9 Bacterial Vaginitis  #10 Vaginitides, Bacterial  #11 #1 or #2 or #3 or #4 or #5 or #6 or #7 or #8 or #9 or #10  #12 MeSH descriptor: [Vitamin D] explode all trees  #13 25hydroxyvitaminD  #14 25(OH)D  #15 ergocalciferol  #16 cholecalciferol  #17 hydroxycholecalciferol  #18 calcifediol  #19 dihydroxycholecalciferol  #20 25hydroxyergocalciferol  #21 #12 or #13 or #14 or #15 or #16 or #17 or #18 or #19 or #20  #22 #11 and #21 |
